# Defining the optimal cut-point of self-reported ART adherence to achieve viral suppression in the era of contemporary HIV therapy: a cross-sectional study

**DOI:** 10.1186/s12981-021-00358-8

**Published:** 2021-06-26

**Authors:** Emma O’Halloran Leach, Huiyin Lu, Joshua Caballero, Jennifer E. Thomas, Emma C. Spencer, Robert L. Cook

**Affiliations:** 1 SHARC Center for Translational HIV Research, University of Florida, 2004 Mowry Road, Gainesville, 32610 USA; 2 SCHARP, Fred Hutchinson Cancer Research Center, 1100 Fairview Ave N, Seattle, WA 98109 USA; 3 Department of Clinical and Administrative Sciences, College of Pharmacy, Larkin University, 18301 North Miami Avenue, Miami, FL 33169 USA; 4 Division of Disease Control and Health Protection, Florida Department of Health, 4052 Bald Cypress Way, Tallahassee, FL BIN A0932399-1715 USA

**Keywords:** HIV, Viral suppression, Self-reported, Adherence, ART

## Abstract

**Background:**

When considering adherence to antiretroviral therapy (ART) for HIV, many different cut-points are used. The primary goals of this study were to identify a level of self-reported medication adherence that best distinguished HIV viral suppression from non-suppression, and to compare the ability of a single-item and a 3-item adherence questionnaire to predict HIV viral suppression.

**Methods:**

This cross-sectional analysis included 380 persons with HIV (PWH) from the Florida Cohort study who completed a self-reported ART adherence measure within 30-days of having an HIV viral load test. We used Receiver Operating Characteristic (ROC) curve analyses and ROCContrast to compare the ability of a single-item and a 3-item self-reported adherence measure to predict HIV viral suppression (defined as ≤ 200 copies/mL). We used the Youden index and chi square statistics to assess specific cut-points, and repeated the analysis with a different definition of HIV viral suppression (≤ 1000 copies/mL).

**Results:**

The mean percent adherence was 92.4% using the single-item score and 90.4% using the 3-item score; 81.6% had viral suppression. The areas under the curve for the single-item and 3-item adherence measures were generally poor overall and not significantly different from each other (0.589 and 0.580, p = 0.67). The Youden index identified cut-points of 93% and 89% as maximizing the sensitivity and specificity for the single-item and 3-item measures, respectively, whereas a cut-point of 80% on the single-item measure was best able to discriminate those with viral suppression (58% vs. 84%, p < 0.001). Results were similar with viral suppression defined as ≤ 1000 copies/mL.

**Conclusions:**

In this sample of PWH, a single question on medication adherence was as good as a 3-item questionnaire in predicting HIV viral suppression, although neither had good discriminatory ability. A cut-point close to 90% adherence maximized sensitivity and specificity, although viral suppression was very similar for nearly all measures above 80%.

## Background

Medication adherence is a common concern when treating any chronic disease; however, it is particularly important when working with persons infected with human immunodeficiency virus (HIV). Since the introduction of newer antiretroviral therapy (ART), there has been a significant decline in mortality among this population [1, 2]. Patients with HIV are encouraged to take all of their medications strictly as directed because suboptimal adherence can lead to detectable viremia, decreased CD4 counts, viral resistance, higher rates of transmission, earlier death, and an overall poorer quality of life [1, 3–8]. However, it is not reasonable to expect perfect adherence for everyone with HIV. Adherence is often a challenge because of a combination of social factors including the stigma of taking medication in public, side effects, concomitant mental health issues, alcohol and/or drug use, patients’ perspective of the drugs’ efficacy, and complexity of the medication regimen [9, 10].

The level of adherence that is required to achieve HIV viral suppression is no longer clear because with newer antiretroviral treatments lower levels of adherence may still achieve successful immunosuppression [11]. Today, antiretroviral medications carry less severe adverse effects, have longer half-lives, and are more readily available in co-formulated products which can be taken once daily, thereby facilitating adherence. Studies vary when defining the “optimal level of adherence”, with different sources using ≥ 85%, ≥ 90%, or ≥ 95% of pills taken as the threshold level necessary to achieve viral load suppression, [1, 12–14]. It will be helpful to know whether there is a cut-point that clearly distinguishes HIV viral suppression from non-suppression. The optimal cut-point could be one that emphasizes sensitivity (do not want to miss anyone], or specificity (do not want any false positives), or the point that maximizes the sensitivity and specificity of the adherence measure to distinguish the outcome of HIV viral suppression.

Measurement of medication adherence is a challenge, and self-report is often used by both researchers and clinicians. Some measures of self-reported adherence use multiple items, whereas others use a single-item such as a visual analog scale [11, 15, 16]. It is not clear whether additional adherence assessment items will improve the ability to discriminate HIV viral suppression compared to a single-item measure.

The definition of viral suppression depends on the detectable limit of the assay, with “undetectable” defining viral suppression [17]. Whereas many contemporary test assays have a lower limit of detection of < 40−75 copies/mL, others have a lower limit of < 200 copies/mL, which is the level defined by the Centers for Disease Control and Prevention in 2019. In some international settings where viral load testing is less common, the lower level of detection may be < 1000 copies/mL, which is the definition of treatment failure according to the World Health Organization’s 2016 guidelines for treating HIV infection [19]. Even among persons who are fully adherent to therapy, occasional low level viremia (50–1000 copies/mL] can occur and does not indicate a higher risk of treatment failure [20]. Therefore, the ability of a medication adherence to predict viral suppression could vary depending on which definition of viral suppression is used (e.g. < 200 copies/mL vs. 1000 copies/mL).

The objectives of this study were: 1) to identify a threshold level of self-reported medication adherence that would most consistently distinguish viral suppression (≤ 200 copies/mL) from non-suppression, and 2) to determine whether a single-item or 3-item self-reported medication adherence measure was most strongly predictive of HIV viral suppression. We also sought to determine if the results would be similar if viral suppression was defined as ≤ 1000 copies/mL.

## Methods

### Study design

The data for this cross-sectional study were collected through the Florida Cohort Survey, a survey given to over 900 people living with HIV across the state of Florida [21]. All participants provided written informed consent. The research procedures were approved by IRBs at the University of Florida, Florida International University, and the Florida Department of Health. The survey included questions on demographics, medical history, medication regimen, mental health, drug and alcohol use, and other factors (questionnaire available at http://sharc-research.org/research/flcohort/). Participants completed the survey privately. Researchers obtained additional clinical information, including HIV viral load, from participants’ medical records and the Florida Department of Health. Survey responses and lab values were deidentified and double data entry was used to maximize the accuracy of the data. Participants were given $25 gift cards as compensation.

### Population and inclusion criteria

Any person over the age of 18 with HIV was eligible to participate. Most of the participants were recruited through public health clinics that provide HIV care; however, some were also recruited through community centers across the state of Florida. Participants were only included in this analysis if HIV-positive status was confirmed by documentation in the medical record, they answered all three questions necessary to determine the two adherence measures, they had received HIV treatment for at least 12 months, and they had HIV viral load results within 30 days of taking the survey. We only included people in treatment for at least one year to narrow down our sample to those who likely had a more stable medication regimen and more stable viral suppression. We only included those who had viral loads drawn within 30 days of taking the survey so that the viral load would most accurately reflect 30-day self-reported adherence.

### Measures

Self-reported adherence was measured in two ways. The first was one simple question from the survey: “In the last 30 days, on how many days did you miss at least one dose of any of your HIV medicine?” Participants wrote in a number of days between 0 and 30. From this, the “single-item self-reported adherence” was calculated for the previous month using this formula:

1 $$\frac{{\left[ {30 - \left( {numberofnon-adherentdays} \right)} \right] \times 100}}{{30}} = One - itemAdherence\%$$

The second way adherence was calculated was through a 3-question summed measure, based on the self-report measure shown by Wilson et al. in 2016 to be valid compared to electronic adherence measures [22]. The questions used to calculate this “3-item self-reported adherence” included

(1) “In the last 30 days, on how many days did you miss at least one dose of any of your HIV medicine?” (write in number of days, 0–30); (2) “In the last 30 days, how well did you do at remembering to take all your prescribed HIV medication?” (excellent, very good, good, fair, poor, very poor); and (3) “In the last 30 days, how often did you take your HIV medication as directed?” (always, almost always, usually, sometimes, rarely, never). In our study dataset, the standardized Cronbach Coefficient Alpha for these three questions was α = 0.80. In order to combine the results of these three questions, the answers to each were converted to a 100-point scale and averaged together to give the final 3-item adherence score. Our scoring of the 3-item questionnaire was similar to the raw scoring approach used by Wilson et al. as we did not include their calibration based on electronic monitoring [22].

For our primary analyses, HIV viral suppression was defined as ≤ 200 copies/ml, which is consistent with the Centers for Disease Control and Prevention’s definition [18]. In addition, we repeated the analyses using a definition of ≤1000 copies/mL.

### Analyses

We determined the average (mean) level of adherence with the single-item and 3-item scales. To better assess the distribution of adherence, we created multiple categories for the proportions of self-reported adherence for both the single-item and 3-item adherence measure (0–75, > 75–80, > 80–85, > 85–90, > 90–95, and > 95–100%). We then determined the proportion of persons with HIV viral suppression (≤ 200 copies/mL) in each category, and used the Chi-square test to assess the statistical significance of any differences in HIV viral suppression that were observed above vs. below any specific cut-point. To assess the adherence measure as a whole, we used simple Logistic regression analysis to determine whether the level of adherence (treated as an ordinal variable) was significantly associated with HIV viral suppression.

Receiver Operating Characteristic (ROC) curves were constructed for the outcome of HIV viral suppression (≤ 200 copies/mL) and the predictors being different levels of adherence as assessed on either the single-item or 3-item adherence score. The area under the curve (AUC) was used to assess the abilities of the single-item and 3-item self-reported adherence measures to predict viral suppression. To compare the two ROC curves, we used SAS PROC LOGISTIC with the ROCCONTRAST statement, and considered p < 0.05 to be statistically significant. A Youden index (J) was applied to each of the ROC curves to identify the adherence percentage cut point that maximized the sensitivity and specificity of predicting viral suppression [23, 24]. These analyses were repeated with a different definition of HIV viral suppression (≤ 1000 copies/mL).

## Results

### Demographics

After applying the inclusion criteria, the sample size included 380 persons living with HIV. The majority were male (63.4%), over the age of 45 years (62.9%), unemployed or unable to work (73.5%), drank alcohol (69.2%), and used drugs in the last year (60.4%). About one third had less than a high school education, another third had a high school diploma or equivalent, and the last third had higher than a high school education. About half were Black, non-Hispanic (55.0%), about a quarter were white, non-Hispanic (23.4%), about a fifth were Hispanic (18.4%), and the remainder of people identified as another race or ethnicity. Most were either not depressed or minimally to mildly depressed (71.5%) (see Table 1).

**Table 1:** Demographic characteristics (N = 380), percentages in various groups

Characteristic	Percent (%)
Sex at birth	
Male	63.4
Female	36.6
Age	
18–34	18.2
35–44	18.9
45–54	36.3
>=55	26.6
Race	
White, not hispanic	23.4
Black, not hispanic	55.0
Hispanic	18.4
Other	3.2
Education	
<High school	33.6
High school diploma or equivalent	31.5
>High school	34.9
Employment	
Employed	26.5
Unemployed	25.9
Unable to work/Disabled	47.6
Depression^a^	
1–4, None-minimal	38.4
5–9, Mild	33.1
10–14, Moderate	14.8
>=15, Moderately severe or severe	13.7
Alcohol use	
Not heavy drinking^b^	60.6
Heavy drinking ^c^	8.4
No drinks in the past year	22.9
Never have drank any alcohol	8.1
Drug use in the past 12 months	
Yes	60.4
No	39.6

^a^Depression score based on eight item Patient Health Questionnaire depression scale (PHQ)-8(41)

^b^Not heavy drinking defined as 1–14 drinks/week for men and 1–7 drinks/week for women

^c^Heavy drinking defined as >14 drinks/week for men and > 7 drinks/week for women

### Reported adherence and viral suppression

The percentage of adherence was high overall, with a mean (standard deviation) of 92.4% (19.0) using the single-item score and slightly lower mean of 90.4% (15.1) using the 3-item score. Most participants had current HIV viral suppression, with 81.6% achieving viral suppression at ≤ 200 copies/mL and 90.3% at ≤ 1000 copies/mL. Overall, the proportion of self-reported adherence was significantly associated with HIV viral suppression (≤ 200 copies/mL) for both the single-item (OR = 6.41, 95% CI: 2.11–19.44) and the 3-item (OR = 1.02, 95% CI: 1.01–1.04) measures.

**Fig. 1:**
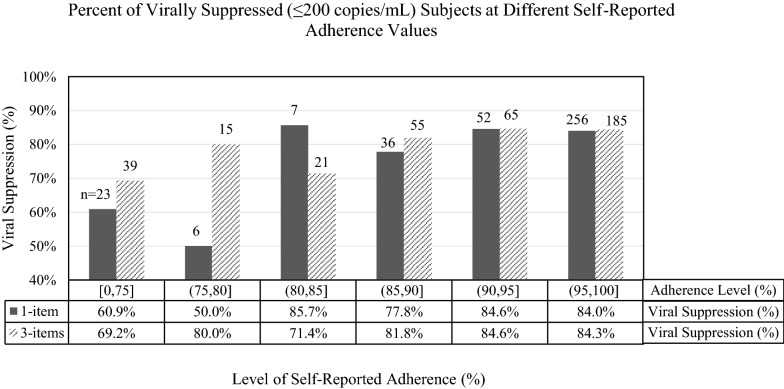
Graph of the proportion of virally suppressed subjects at different levels of single-item and 3-item self-reported adherence. Viral suppression is defined as ≤200 copies/mL. [0,75] indicates the group was between 0 and 75% adherent based on the single-item or 3-item scale. Parentheses indicate a value is not included and brackets indicate a value is included (e.g. (90,95] does not include 90 and does include 95). The percentages describe the proportion of viral suppression for each level of self-reported adherence

When considering specific cut-points, almost all of the participants reported greater than 75% adherence (94.0% based on single-item; 89.7% based on 3-item). Far fewer reported greater than 95% adherence (67.4% based on single-item; 48.7% based on 3-item). Figure 1 details the proportion of virally suppressed participants at different self-reported adherence values based on the single-item and 3-item self-reported adherence scales. For the single-item scale, the proportion of persons with HIV viral suppression appeared to be fairly similar for all values above 80%, but lower in those with less than 80% (84% vs. 58%, p < 0.001). For the 3-item score, no specific cut-point appeared to provide a clear distinction between those with or without HIV viral suppression (Fig. 1). Results were similar using a cut-point for HIV viral suppression of ≤1000 copies/mL (data not shown).

### Receiver operating characteristic (ROC) curves

**Fig. 2:**
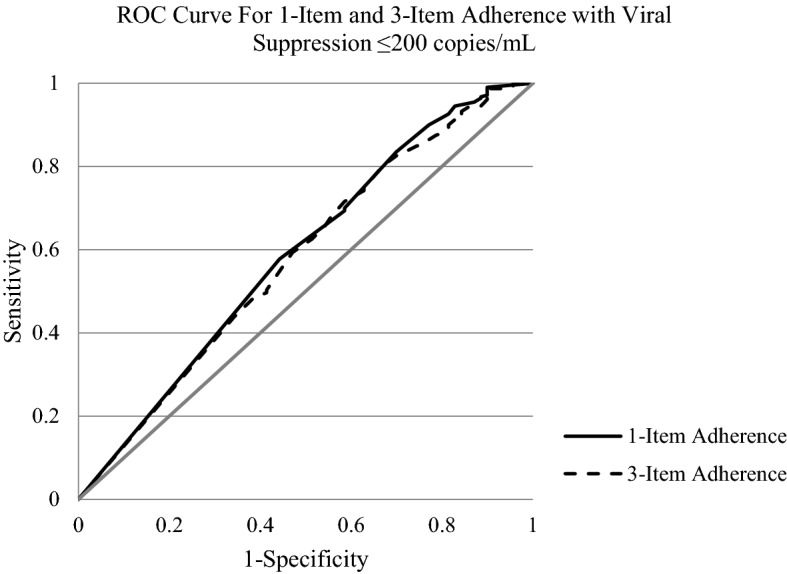
ROC curve showing single-item adherence and 3-item adherence with viral suppression ≤200 copies/mL. single-item AUC is 0.589 and 3-item AUC is 0.580

**Fig. 3:**
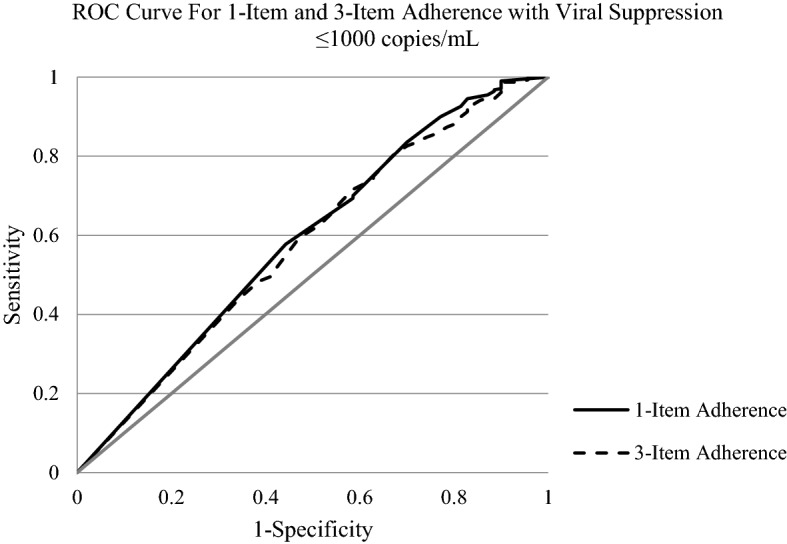
ROC curve showing single-item adherence and 3-item adherence with viral suppression ≤ 1000 copies/mL. single-item AUC is 0.631 and 3-item AUC is 0.653

When viral suppression was defined as ≤ 200 copies/mL, the AUC for the ROC curves for the single-item and 3-item adherence measures were nearly identical (0.589 vs. 0.580, p = 0.67, see Fig. 2). The AUC for the single-item vs. 3-item adherence measures were also nearly identical when using a definition of HIV viral suppression of ≤ 1000 copies/mL (0.631 vs 0.653, p = 0.47, see Fig. 3).

Based on the Youden index of the ROC curves, the optimal adherence level to maximize both sensitivity and specificity was 93% for the single-item adherence and 89% for the 3-item adherence for viral suppression ≤ 200 copies/mL. For viral suppression defined as ≤1000 copies/mL, the optimal cut point to maximize the sensitivity and specificity was 93% for the single-item adherence and 87% for the 3-item adherence.

## Discussion

The primary goals of this study were to identify a level of self-reported medication adherence that best distinguished HIV viral suppression from non-suppression, and to compare the ability of a single-item and a 3-item adherence questionnaire to predict HIV viral suppression. Overall, examination of the ROC curves suggested that there is no cut-point of self-reported adherence that clearly distinguished HIV viral suppression from non-suppression. We also found that a single-item adherence question performed equally well as a 3-item self-reported adherence assessment.

### Threshold levels of adherence to predict viral suppression

Historically in the literature, 95% adherence has been used for this threshold [12, 17]. However, with newer, more potent ART, lower levels of adherence have proven to be adequate. One way to define an optimal cut-point is to choose the point indicated by the Youden index, which determines a point on the ROC curve that maximizes both the sensitivity and specificity. According to these criteria, the optimal cut-point was 93% for the single-item question and 87% for the 3-item questionnaire. However, as noted in Fig. 1, the proportion of people who were virally suppressed was nearly identical for all persons reporting over 80% adherence on the single-item measure, and much lower for those reporting <80% adherence. Therefore, in our data, an 80% self-reported adherence rate (equivalent to adherence on 24 out of the past 30 days) might best distinguish persons with or without viral suppression.

While the percentage of adherence was statistically significantly associated with HIV viral suppression in logistic regression analyses, the AUC for the ROC curves suggests that the overall ability to discriminate HIV viral suppression is poor. An AUC of greater than or equal to 0.70 is considered fairly predictive [25]; however, the AUCs in our sample were < 0.60 for both of the adherence measures and HIV viral suppression ≤200 copies/mL. These results are similar to another study which found self-reported adherence did not significantly predict viral suppression defined as < 400 copies/mL [26]. These conclusions are important because self-reported adherence is commonly used as an outcome measure in the literature [15]. However, self-reported ART adherence does not appear to be a great predictor of viral suppression in this sample.

### Comparing single-item and 3-item adherence and viral suppression

Another goal of the study was to compare the ability of the single-item and 3-item self-reported adherence measures to predict viral suppression. We found that the predictive ability of a single item, “In the last 30 days, on how many days did you miss at least one dose of any of your HIV medicine?”, was essentially the same as a 3-item measure that included this item plus two additional items that asked about trouble remembering to take medication and taking medication off schedule. As seen in Figs. 2 and 3, the AUC’s of the ROC curve analysis demonstrated that the discriminatory ability of both self-reported measures was nearly identical whether HIV viral suppression was defined as ≤ 200 copies/mL or ≤ 1000 copies/mL. A single-item measure is much simpler and easier to administer compared to the three-item measure, and our data provide increased confidence for studies that used only a single item to measure medication adherence. Other methods to assess self-reported adherence include the AIDS Clinical Trials Group (ACTG) questionnaire which includes multiple items that assess not only if participants have missed doses (e.g., days, weekend, month), but accounts for how closely they followed the schedule and directions [27, 28]. Another single-item measure that may be considered is the visual analog scale which asks patients to estimate their adherence on a line, and which has generally been able to produce similar results as multi-item self-reported assessments [15].

It is unclear whether more objective methods to measures medication adherence would have been more strongly associated with viral suppression. While electronic medication monitoring systems to supplement self-reported measures are available, they are expensive and impractical (e.g. bottle incompatibility, misclassification) in a clinical non-research setting [11]. Pill counts could be used, but the use of multiple pill boxes, or patients taking out of the bottle (to hide status) may create discrepancies [29]. Additionally, pill counts may be time consuming and perceived as intrusive [30]. Ingestible biosensors are an emerging technology currently being studied as a potential method to monitor adherence [31]. There are also studies that have used plasma, urine or hair tests to measure ART concentrations and determine adherence [32–34]. Future studies could prospectively compare these more objective measures to self-reported measures to see how well they predict viral suppression and to further determine the optimal level of adherence.

## Limitations

Our study does have some limitations. Our outcome measure of HIV viral suppression was obtained on a single day within a given month, and it is unknown if the measured viral load was representative of their viral suppression for the entire month that corresponded with our adherence measure. We attempted to control for this to an extent by only including participants who had been on ART for at least a year and who had a viral load test done within 30 days of the self-reported adherence assessment. Our viral load results could only consistently evaluate a lower limit of detection of HIV viral load of ≤200 copies/mL, while the level of detection for newer assays generally ranges from < 20 to 75 copies/mL [30]. While one long-term study found an increased risk of treatment failure with persistent low-level viremia at >50 copies/mL, another study found that HIV viral levels of < 200 copies/mL and < 50 copies/mL had the same predictive value for subsequent viral rebound [35, 36]. We did not consider the specific drug regimen of our participants, and it is possible that different levels of adherence are needed to achieve viral suppression with different treatment regimens. While older treatment options required over 95% adherence to be effective, newer treatment options allow persons to achieve viral suppression even if adherence is close to 80%, [37] and our results are consistent with this conclusion. Future studies may need to assess specific medication regimens and their effects on adherence and viral suppression.

## Conclusions

In conclusion, no specific cut-point for the percentage adherence of ART could clearly distinguish HIV viral suppression of ≤ 200 copies/mL or ≤ 1000 copies/mL. The ROC curves demonstrated that self-reported ART adherence was statistically significantly associated with HIV viral suppression, however, the overall ROC scores of < 0.70 suggest that the true discriminatory ability is relatively poor. While a cut-point around 90% adherence maximized the overall sensitivity and specificity, our data suggest a noticeable drop-off in HIV viral suppression for persons reporting < 80% adherence. A single question about the number of days of missed medication in the past month was as predictive as a 3-item questionnaire.

## Data Availability

A de-identified dataset for the Florida Cohort is available for sharing. The process and policies to request and obtain such a dataset are described on the SHARC web site at http://sharc-research.org/get-involved/submit-a-concept/.

## Abbreviations

ACTG: AIDS Clinical Trials Group
AIDS: Acquired immunodeficiency syndrome
ART: Antiretroviral therapy
AUC: Area under the curve
HAART: Highly active antiretroviral therapy
HIV: Human immunodeficiency virus
PWH: Persons with HIV
ROC: Receiver operating characteristic
